# Ki-67 is required for maintenance of cancer stem cells but not cell proliferation

**DOI:** 10.18632/oncotarget.7057

**Published:** 2016-01-28

**Authors:** Justin Cidado, Hong Yuen Wong, D. Marc Rosen, Ashley Cimino-Mathews, Joseph P. Garay, Abigail G. Fessler, Zeshaan A. Rasheed, Jessica Hicks, Rory L. Cochran, Sarah Croessmann, Daniel J. Zabransky, Morassa Mohseni, Julia A. Beaver, David Chu, Karen Cravero, Eric S. Christenson, Arielle Medford, Austin Mattox, Angelo M. De Marzo, Pedram Argani, Ajay Chawla, Paula J. Hurley, Josh Lauring, Ben Ho Park

**Affiliations:** ^1^ The Sidney Kimmel Comprehensive Cancer Center, The Johns Hopkins University School of Medicine, Baltimore, MD, USA; ^2^ Cardiovascular Research Institute, University of California San Francisco, San Francisco, CA, USA; ^3^ Departments of Physiology and Medicine, University of California San Francisco, San Francisco, CA, USA; ^4^ The Whiting School of Engineering, Department of Chemical and Biomolecular Engineering, The Johns Hopkins University, Baltimore, MD, USA; ^5^ Present address: Oncology iMED, AstraZeneca, Waltham, MA, USA; ^6^ Present address: Roche Sequencing, San Jose, CA, USA

**Keywords:** Ki-67, cancer stem cells, proliferation, clonogenicity, tumorigenicity

## Abstract

Ki-67 expression is correlated with cell proliferation and is a prognostic marker for various cancers; however, its function is unknown. Here we demonstrate that genetic disruption of Ki-67 in human epithelial breast and colon cancer cells depletes the cancer stem cell niche. Ki-67 null cells had a proliferative disadvantage compared to wildtype controls in colony formation assays and displayed increased sensitivity to various chemotherapies. Ki-67 null cancer cells showed decreased and delayed tumor formation in xenograft assays, which was associated with a reduction in cancer stem cell markers. Immunohistochemical analyses of human breast cancers revealed that Ki-67 expression is maintained at equivalent or greater levels in metastatic sites of disease compared to matched primary tumors, suggesting that maintenance of Ki-67 expression is associated with metastatic/clonogenic potential. These results elucidate Ki-67's role in maintaining the cancer stem cell niche, which has potential diagnostic and therapeutic implications for human malignancies.

## INTRODUCTION

Ki-67 is a protein that is widely used as a marker for cell proliferation, and its increased expression in human cancer specimens generally denotes an aggressive phenotype. Molecular cloning of the Ki-67 cDNA led to its identification as a large nuclear protein, and further studies revealed its nuclear localization during cell cycling [1] as well as its association with proteins involved with cell division [2]. Ki-67 has often been used as a prognostic marker for a number of human malignancies, either as a sole criterion (reviewed in [3]) or as part of a panel of genes to further define risk of recurrence for a given cancer subtype [4]. Despite its widespread use in clinical pathology and oncology, there is a paucity of studies characterizing Ki-67's cellular function.

Given its past association with cell proliferation, it has been speculated that absence of Ki-67 would result in cell lethality or a permanent state of arrest [1]. However, this theory remains controversial, as normal tissues with viable proliferating cells have been described to have low to absent levels of Ki-67 expression [5, 6]. Insights from functional studies of Ki-67 have been limited in part due to the large nature of the Ki-67 protein, its extreme sensitivity to protease degradation, and its short half-life, all of which hinder molecular analyses. Studies using anti-sense RNA and RNA interference (RNAi) suggest that gene knockdown of Ki-67 leads to reduced cell proliferation [7, 8]. However, due to the incomplete nature of gene knockdown and potential off-target effects [9], these data are not definitive. Furthermore, cell lethality was not addressed in these systems. Regardless, the fact that many aggressive cancers have high expression of Ki-67 relative to normal and quiescent cells suggests that Ki-67 might be an attractive target for cancer therapy if its role as a driver of carcinogenesis were validated.

## RESULTS

### Gene knock out of Ki-67 is not lethal in human cells

**Figure 1:**
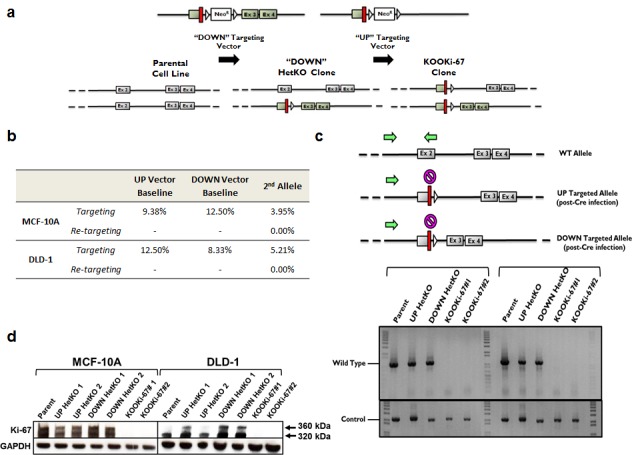
Gene targeting of Ki-67 **a**. The targeting constructs consist of a neomycin resistance gene flanked by loxP sites and homology arms. Two promoterless adeno-associated virus (AAV) targeting vectors were synthesized, differing only in their 3′ homology arms termed “UP” and “DOWN” for relative 5′ and 3′ positions, respectively. Shown are constructs before Cre mediated excision. Red bars denote “STOP” sequences in all three reading frames. **b**. Targeting frequencies of individual UP and DOWN vectors in MCF-10A and DLD-1 cells. **c**. PCR was performed to confirm gene targeting of *MKI67* alleles. The reverse primer was designed to anneal to a genomic sequence that is deleted (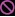) with either vector. The absence of a PCR product for the KOOKi-67 clones indicates both alleles have been properly targeted. A separate control PCR across the first coding exon was performed to ensure the presence of gDNA in all samples. Green arrows denote primers used in PCR screens. **d**. Western blot for Ki-67 protein in parental, HetKO and KOOKi-67 cell lines using GAPDH as an internal loading control.

We asked whether Ki-67 was necessary for cell viability and proliferation. To test this hypothesis, we deleted Ki-67 in two distinct human cell lines: MCF-10A and DLD-1. MCF-10A is a non-tumorigenic, spontaneously immortalized, genetically stable human breast epithelial cell line, whereas DLD-1 is a human colorectal cancer cell line. The targeting vectors were designed to insert premature nonsense (STOP) mutations into the first coding exon (exon 2) of the *MKI67* gene via AAV-mediated gene targeting [10], thus disrupting all sense open reading frames (Figure 1a). Two rounds of gene targeting were necessary since MCF-10A and DLD-1 cells are diploid for *MKI67*. For proof of non-lethality (see below), we employed two targeting vectors that had identical 5′ homology arms but differed in their 3′ homology arms to prevent “re-targeting” while allowing for selective targeting of independent alleles.

Through successful gene targeting we found that knockout of Ki-67 is not a lethal event. Although random changes within single cells could theoretically circumvent lethality caused by disruption of the second allele, such events would be predictably rare. In contrast, the targeting frequency of a non-lethal event would be expected to be approximately 50% of the original targeting frequency since only one of two wildtype alleles remains. We used two distinct targeting vectors, termed “UP” and “DOWN”, which refers to the relative upstream and downstream positions of their 3′ homology arms (Figure 1a). These vectors generated heterozygous knockout (HetKO) clones with a targeting frequency of ∼ 8% to 12% for each vector in both cell lines used (Figure 1b). Homozygous null clones were generated by using the UP vector in DOWN-targeted HetKO clones. Using this strategy, the UP vector was predicted to target only the remaining wildtype allele since the corresponding 3′ homology arm sequence has been deleted in the DOWN-targeted allele (Figure 1a). As shown in Figure 1b, generation of Ki-67 null cells, termed KnockOut Of Ki-67 (KOOKi-67), was observed at a targeting frequency of ∼ 4% to 5%, approximately half of that observed for the generation of HetKO. We also detected no re-targeting events, further demonstrating the specificity of this method for the remaining wildtype allele. Gene targeting for Ki-67 null cells was assessed using PCR of gDNA, and two independently established clones for both MCF-10A and DLD-1 cell lines were isolated using PCR screening (Figure 1c). Absence of Ki-67 protein in KOOKi-67 cell lines was then confirmed via western blot (Figure 1d). These results demonstrate that homozygous gene disruption of Ki-67 is not a rare, artifactual event, but is in fact compatible with cell viability and proliferation.

### Knock out of Ki-67 does not affect cell proliferation or chromosomal instability

**Figure 2:**
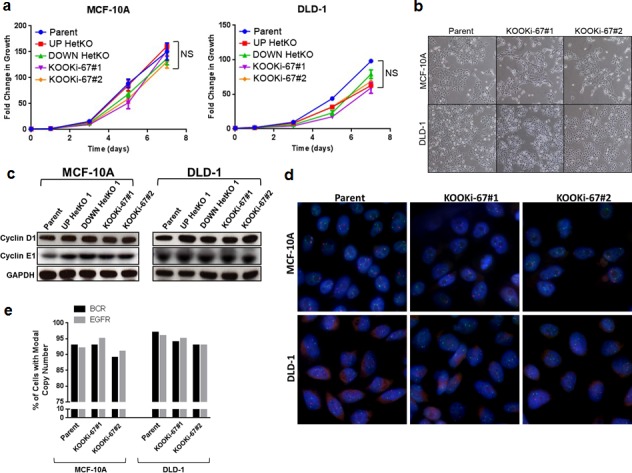
Loss of Ki-67 does not affect cell proliferation in bulk culture or alter morphology and does not induce chromosomal instability **a**. MCF-10A and DLD-1 isogenic cell lines were seeded at 10^3^ cells per well in 96-well plates to measure cell growth over a 7-day time course via CellTiter-Glo. NS = not significant. **b**. Representative phase contrast micrographs displaying normal cell morphology for MCF-10A and DLD-1 parental and KOOKi-67 clones (200x). **c**. Western blot for cyclin D1 and cyclin E1 in parental, HetKO and KOOKi-67 cell lines using GAPDH as an internal loading control. **d**. FISH was performed on parental and KOOKi-67 clones from MCF-10A and DLD-1 cells to assess for chromosomal instability. Cells were probed for EGFR (red) and BCR (green) loci, with representative experiments displayed. **e**. The modal copy number (N = 2 for both probes) was determined by counting 200 cells from each cell line, and chromosomal instability assessed as cells deviating from the modal copy number.

Based on these results, we next investigated whether knockout of Ki-67 conferred a growth disadvantage for the KOOKi-67 clones. Initial characterization of cell proliferation kinetics revealed no obvious disadvantage (Figure 2a), and no overt differences in morphology were noted (Figure 2b). Similarly, no clear changes in cell cycle proteins such as cyclin D1 and cyclin E1 were observed between KOOKi-67 cells and controls (Figure 2c). Given previous studies linking Ki-67 to higher-order chromatin structure [11], we also asked whether the loss of Ki-67 could result in chromosomal instability (CIN). However, FISH analysis with two gene-specific probes showed no evidence of CIN (Figure 2d and 2e). These results support that Ki-67 is not necessary for cell proliferation and does not appear to affect CIN.

### Knock out of Ki-67 decreases *clonogenic* proliferation *in vitro* and *in vivo*

**Figure 3:**
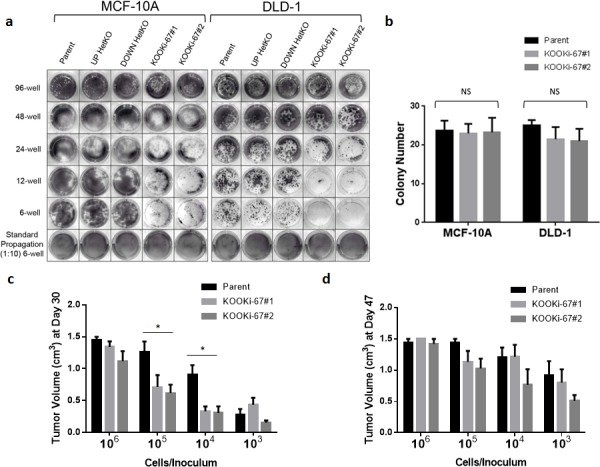
Ki-67 null cells have decreased clonogenic proliferation *in vitro* and *in vivo* **a**. MCF-10A and DLD-1 isogenic cell lines were seeded at 10^3^ cells per well in 96-well, 48-well, 24-well, 12-well, and 6-well plates under standard growth conditions, grown for 10 days, and stained with crystal violet. **b**. Parental and KOOKi-67 clones were also seeded at 75 cells per well in 6-well plates and grown for 10 days under standard conditions, at which time the number of colonies per well were counted. NS = not significant. **c**. DLD-1 parental and KOOKi-67 clones were injected subcutaneously into the flank of athymic female nude mice at the varying densities shown. Tumor volume was measured once per week until the tumor reached a maximal volume of 1.5 cm^3^. The average tumor volumes are plotted to display differences between parental and KOOKi-67 cells at each concentration used for injection at Day 30 and **d**. Day 47. * p < 0.05. All experiments are representative of at least three experiments using multiple replicates within each experiment.

In generating KOOKi-67 cell lines, we noticed for both MCF-10A and DLD-1 that recovery of single cell clones after Cre-loxP excision took considerably longer compared to the generation of HetKO cells and past gene targeting experiments performed in our lab. Thus, we hypothesized that Ki-67 may be specifically affecting single cell *clonogenic* proliferation, that is, the ability to grow from a single cell in the absence of other neighboring cells. We initially tested this hypothesis by seeding the same number of cells (10^3^) in various sized tissue culture plates (6, 12, 24, and 48 wells). As depicted in Figure 3a, there was a perceptible difference in colony size, which became more apparent with decreasing cell density. Enumeration of colonies grown in six well plates demonstrated that colony number seemed minimally affected (Figure 3b), and only the size of colonies, that is proliferation of individual clones, was decreased in KOOKi-67 cells. Since potential artifacts from satellite colonies can arise in these assays, we confirmed these results in single cell limiting dilution assays. We seeded five 96-well plates per cell line with a calculated density of 0.5 cells per well, allowing us to count single cell colonies per plate. For both the MCF-10A and DLD-1 cells, the KOOKi-67 clones contained approximately the same number of colonies as the parental cell line but were noticeably smaller in colony size (Supplemental Figure 1). We could only score these colonies at a time point approximately two to three weeks after their parental counterparts yielded visible clones, reaffirming our initial observations seen with the generation of KOOKi-67 clones. These results show that knockout of Ki-67 does not directly affect total number of colonies, nor proliferation in bulk culture, but does result in decreased clonogenic proliferation.

We then asked whether lack of Ki-67 would also affect clonogenic proliferation *in vivo*. Since MCF-10A cells are non-tumorigenic, only DLD-1 clones could be used for these experiments. Ten-fold serial dilutions (10^6^ to 10^3^ cells) of each DLD-1 clone were injected subcutaneously into athymic nude mice, and tumors were allowed to grow until reaching a maximum volume of 1.5cm^3^. Similar to the *in vitro* assays, we found that parental DLD-1 and KOOKi-67 clones had comparable rates of tumor growth at the highest concentration of cells used for the inoculum. However, at the next lowest dilution, parental cells still achieved maximal growth in the 30 day assay, whereas KOOKi-67 clones had significantly less growth (Figure 3c). This was also observed at the 10^4^ cell inoculum, whereas 10^3^ cells per injection resulted in significantly decreased tumor formation for both parental and KOOKi-67 clones. Analogous to the *in vitro* data, KOOKi-67 clones did eventually achieve similar maximal xenograft volumes at day 47 (Figure 3d). These *in vivo* results recapitulated our *in vitro* data, demonstrating that sparse seeding leads to decreased clonogenic proliferation.

### Knock out of Ki-67 affects stem cell markers, but protein and gene expression profiles are minimally altered

**Figure 4:**
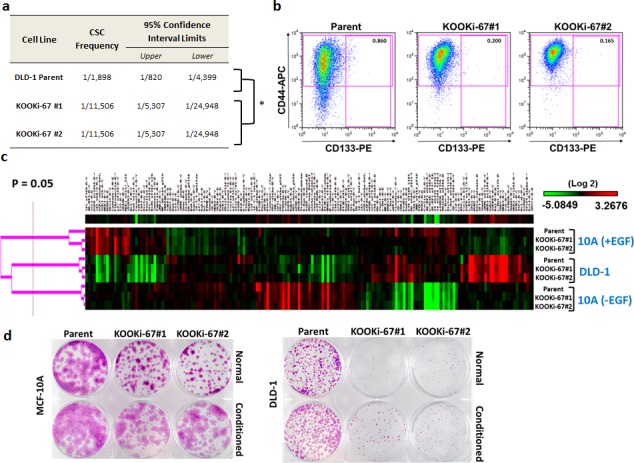
Cancer stem cell frequency and markers are decreased in KOOKi-67 clones without global changes in protein expression **a**. Stem cell frequency was calculated as described in the text using extreme limiting dilution analysis (ELDA); p = 0.000029. **b**. Flow cytometry was performed to assess the population of CD133^+^CD44^+^ cells in DLD-1 parental and KOOKi-67 cells. Representative results are shown from three independent experiments. **c**. MCF-10A and DLD-1 parental and KOOKi-67 clones were subjected to RPPA analysis. DLD-1 cells were grown in standard full growth conditions while MCF-10A cells were grown in both full growth (+EGF) and growth arrest (-EGF) condition. **d**. MCF-10A and DLD-1 parent cell lines were grown to 70 – 80% confluency, and conditioned media was filtered through a 0.2 μm filter. Parent and KOOKi-67 cell lines were seeded at 10^3^ cells per well in 6-well plates and grown over 10 days in either normal growth media or a 1:1 mixture of conditioned:normal growth media.

It has been postulated that solid tumors contain a subpopulation of cells, termed tumor initiating cells or cancer stem cells (CSCs), which are required for engraftment in various mouse models. Although most studies characterize limiting numbers of CSCs for their ability to form tumors within a defined time period, recent reports have suggested that reduced numbers of CSCs used for inoculations can still lead to tumor formation but with longer latency [12], consistent with our *in vivo* results. Based on prior studies, statistical software has been developed to better quantify stem cell populations based upon limiting dilution experiments [13]. Using these tools, we calculated a frequency of 1 CSC in 1,898 total cells for parental DLD-1 (Figure 4a, Supplemental Table 1). In contrast, both KOOKi-67 clones had a calculated frequency of 1 CSC in 11,506 total cells. We therefore asked whether DLD-1 and KOOKi-67 clones differentially expressed CSC cell surface markers, analyzing clones by flow cytometry to assess the percentage of cells with the known colorectal CSC markers CD133 and CD44. Prior reports indicate that CD133^+^CD44^+^ cells consistently form xenografts [14], and high levels of double-positive cells are a strong indicator for worse disease-free survival and increased risk of recurrence when identified in primary tumors [15]. Consistent with this notion, a high proportion of CD133^+^CD44^+^ cells have been shown to be present in liver metastases, suggesting clonal selection from a CSC population in the primary tumor [16]. As shown in Figure 4b, we found that CD133^+^CD44^+^ cells comprised 0.860% of the parental DLD-1 cell population, whereas the percentage of CD133^+^CD44^+^ cells in KOOKi-67 cell lines was reduced to approximately 0.200% and 0.165%. These data suggest that loss of Ki-67 can negatively impact the CSC population as measured by CD133 and CD44 expression.

Given the change in CD133 and CD44 expression seen with Ki-67 knock out, we then asked if there were any overt differences in protein expression between parental and KOOKi-67 cells. We subjected the cell lines to a proteomic analysis using reverse phase protein arrays (RPPA). For this analysis we also included lysates from growth arrested MCF-10A cells, accomplished through removal of exogenous EGF (17), to compare any protein expression changes related specifically to proliferation. As seen in Figure 4c (protein list shown in Supplemental Figure 2), the overall protein expression patterns remained similar between parental cells and Ki-67 null derivatives, with differences seen between EGF versus no EGF conditions, and between MCF-10A and DLD-1 cells, but no specific consistent changes related to lack of Ki-67. Thus, knock out of Ki-67 did not appear to globally alter protein expression.

Although RPPA analysis did not reveal any noticeable differences in KOOKi-67 cells, we hypothesized that cytokine and growth factor profiles may be affected with knock out of Ki-67 since it is well-known that cell lines that do not yield robust colonies in limiting dilution assays can often be “rescued” by conditioned media. We initially tested this hypothesis by growing parental and KOOKi-67 cells with and without conditioned media. As shown in Figure 4d, conditioned media increased colony size in KOOKi-67 and parental cell lines, but did not fully restore clonogenic growth relative to parental controls. Similar to the RPPA analysis, we found that a focused cytokine and growth factor gene expression array using quantitative real time PCR (qPCR) demonstrated differences between MCF-10A and DLD-1 cells but no appreciable changes that could be attributed to Ki-67 knock out in both cell lines (Supplemental Figure 3). Thus, this limited gene expression analysis did not detect any obvious pathways or genes that could account for the effect of Ki-67 on maintaining the CSC niche.

### The amino terminus containing a forkhead associated (FHA) domain mediates Ki-67's ability to maintain the CSC niche

**Figure 5:**
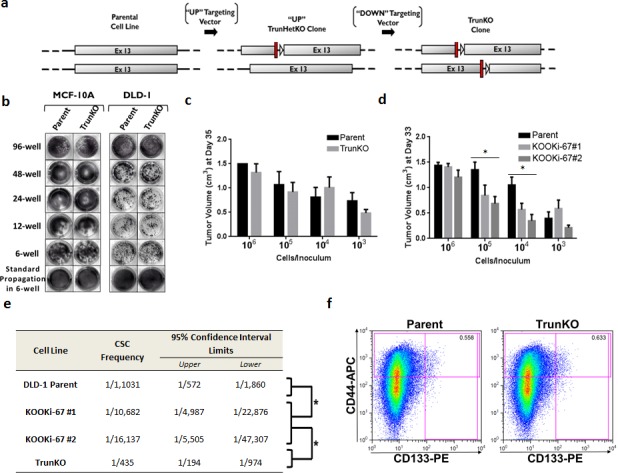
The amino terminus of Ki-67 containing a FHA domain can regulate the cancer stem cell compartment **a**. Schematic of targeting constructs used to create truncated knock outs (TrunKO). Two promoterless adeno-associated virus (AAV) targeting vectors were synthesized, designed to target distinct regions of the Ki-67 repeat domains and termed “UP” and “DOWN” for relative 5′ and 3′ positions, respectively. Shown are constructs after Cre mediated excision. Red bars denote “STOP” sequences in all three reading frames. **b**. MCF-10A and DLD-1 parental and TrunKO cell lines were seeded at 10^3^ cells per well in 96-well, 48-well, 24-well, 12-well, and 6-well plates under standard growth conditions, grown for 10 days, and stained with crystal violet. **c**. DLD-1 parental, TrunKO and KOOKi-67 clones were injected subcutaneously into the flank of athymic female nude mice at the varying densities shown. Tumor volume was measured once per week until the tumor reached a maximal volume of 1.5 cm^3^. The average tumor volumes are plotted to display differences between parental, TrunKO and KOOKi-67 cells at each concentration used for injection. * p < 0.05. **e**. Stem cell frequency was calculated as described in the text using extreme limiting dilution analysis (ELDA); *p < 0.0001. **f**. Flow cytometry was performed to assess the population of CD133^+^CD44^+^ cells in DLD-1 parental and TrunKO cells.

We next attempted to discern which functional domain of Ki-67 might be responsible for modulating the CSC phenotype. Ki-67 has several known domains, including an amino-terminus FHA domain, a large “Ki-67 repeat” domain, and a carboxy-terminus Kon21 domain [17]. We initially created two vectors designed to disrupt the “Ki-67 repeat” domain (Figure 5a) through targeting distinct regions within this domain to prevent re-targeting. A homozygous truncated knockout (TrunKO) was obtained for both MCF-10A and DLD-1 cell lines as demonstrated by PCR screening (Supplemental Figure 4A) and the presence of truncated proteins by western blot (Supplemental Figure 4B). We then asked if these TrunKO clones would have similar effects on clonogenic proliferation and other CSC properties as the KOOKi-67 clones. We first performed clonogenic assays on TrunKO cells where we observed no discernible difference in colony growth between TrunKO and parental controls for both MCF-10A and DLD-1 derived cell lines (Figure 5b). We next tested the DLD-1 TrunKO cells in *in vivo* clonogenicity assays. As shown in Figure 5c, 5d, 5e and Supplemental Table 2, TrunKO cells displayed a similar pattern of cellular dilution, tumorigenicity and calculated CSC frequency as the parental controls. Finally, CSC markers were assessed in DLD-1 TrunKO cells. As shown in Figure 5f, CD133^+^CD44^+^ cells comprised 0.558% of the parental DLD-1 cell population while the percentage of CD133^+^CD44^+^ cells in TrunKO cell lines was roughly equivalent at 0.633%. Together, these data suggest that the FHA domain of Ki-67 is responsible for regulating the CSC population as measured by CD133 and CD44 expression.

### KOOKi-67 cells are more sensitive to chemotherapy, and Ki-67 expression is stable-to-increased in metastatic tumors

**Figure 6:**
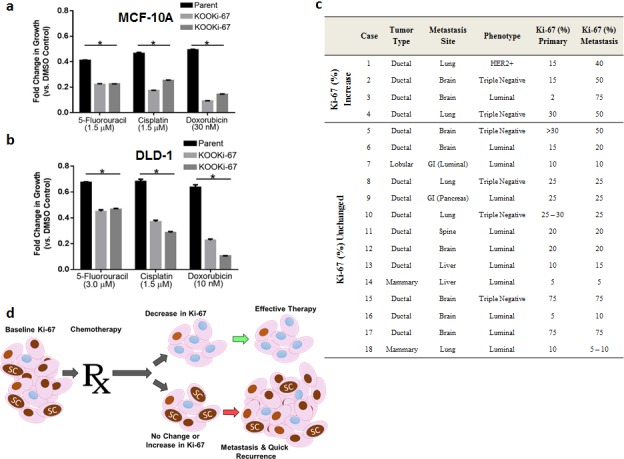
Ki-67 expressing cells are relatively resistant to chemotherapy and persistent Ki-67 expression is present in metastatic tumors **a**. Parental and KOOKi-67 cell lines from MCF-10A and **b**. DLD-1 cells were seeded and treated the following day with chemotherapies and vehicle control as described in Methods. The results are shown as fold change in growth relative to the vehicle control. * p<0.05. Results are representative from three independent experiments. **c**. Ki-67 staining of matched primary and metastatic breast cancers. HER2, human epidermal growth factor 2; GI, gastrointestinal tract. **d**. A proposed model of how changes in Ki-67 after chemotherapy may predict for effective versus ineffective response. Ki-67 positive cells (brown nuclei) mediate the stem cell (SC) niche. Effective chemotherapy dramatically reduces Ki-67 expressing cells, which in turn leads to reduced or eradicated cancer stem cells. Ineffective chemotherapy may reduce the bulk population of cells, but the percentage of Ki-67 expressing cells and subsequently stem cells remain unaffected, resulting in progression and metastases.

A conundrum in oncology is that high Ki-67 expression in cancers is associated with a better response to chemotherapies (predictive marker) yet is also associated with worse disease-free and overall survival (prognostic marker). This predictive-prognostic paradox could be reconciled if Ki-67 expression modulated a CSC-drug resistant population, and therapeutic effectiveness was evaluated not by gross response of tumor shrinkage, but by assessing Ki-67 expression in remaining tumor tissue post chemotherapy. Thus, we hypothesized that if Ki-67 increases a CSC-drug resistant population, KOOKi-67 cells should demonstrate increased sensitivity to chemotherapy agents relative to parental controls in clonogenic assays. As depicted in Figure 6a and 6b, *in vitro* assays with MCF-10A, DLD-1 and KOOKi-67 cells showed that Ki-67 null cells have increased sensitivity to various chemotherapeutic drugs. These results indicate Ki-67 may lead to a persistent drug resistant population and reaffirm that Ki-67 regulates the CSC population.

Cancer metastases can arise due to ineffective therapies. Thus, we reasoned that metastases from cancer patients would have equal or elevated Ki-67 expression compared to corresponding primary lesions from the same patient if Ki-67 expression was indeed modulating a CSC niche, resulting in therapeutic resistance and an increased metastatic potential. We therefore evaluated Ki-67 expression using a cohort of 18 matched primary and metastatic human breast cancers. As seen in Figure 6c, Ki-67 labeling of matched primary and metastatic human breast cancer samples from 18 patients demonstrated an increase in Ki-67 labeling in 4 of 18 cases, while in the remaining cases Ki-67 expression was unchanged. These results are consistent with clinical reports that effective therapies are associated with a decrease in Ki-67 expression (see Discussion) [3, 18] and further support that Ki-67 may be mediating a more aggressive, drug resistant CSC phenotype.

## DISCUSSION

Although Ki-67 has been used for decades as a marker of cell proliferation, there remains a paucity of studies examining its functional significance. Given Ki-67's association with cell proliferation, many have speculated that it is an essential gene and gene disruption or “knock out” would result in a lethal phenotype. However, our study demonstrates that Ki-67 is not necessary for cell survival but reveals an unexpected and unique function of Ki-67 as a regulator of CSC properties. These results have potential implications for cancer biology and therapy. First, since Ki-67 does not affect cellular proliferation as a bulk culture, its clinical importance may be its ability to mediate and maintain a stem cell niche capable of proliferating robustly as a single cell. This property is a well-accepted principle of how solid malignancies metastasize and ultimately cause death. It should be emphasized, however, that we are not suggesting that Ki-67 is a CSC marker, rather that it can functionally regulate the CSC compartment since KOOKi-67 cells can still form tumors; and conversely, MCF-10A cells with high levels of Ki-67 are non-tumorigenic. Second, a drug resistant population in the primary tumor, often attributed to CSCs, can be directly correlated with the percentage of remaining Ki-67 positive cells after neoadjuvant therapies [3, 18]. This strongly suggests that tumors with the same or higher percentage of Ki-67-expressing cells after treatment contain drug resistant stem cells capable of seeding distant sites and forming clinically relevant metastases in a short time frame. Our *in vitro* data showing that loss of Ki-67 renders cells more sensitive to chemotherapy and our analysis of patient samples support this notion. Third, our gene targeting results strongly suggest that Ki-67 may serve as a viable target for cancer therapy, since decreasing the stem cell compartment of solid tumors would abrogate the ability of these cancers to quickly proliferate as single cell metastases, and may help eradicate an inherently drug resistant population of cancer cells. Therefore, a targeted therapy against Ki-67 may lead to a highly effective treatment for many aggressive human cancers. One could envision that anti-Ki-67 therapies coupled with existing chemotherapies may be more effective in tumors with high Ki-67 expression.

Although our studies support that Ki-67 is not required for cell proliferation but functions as a regulatory factor for CSCs, the precise mechanism of how this occurs is unknown. A proteomic and mRNA analysis did not reveal obvious and consistent differences between KOOKi-67 cells and their parental counterparts. However, genetic functional analysis did demonstrate that the amino terminus of Ki-67, containing a FHA domain, is responsible for regulating the CSC population. FHA domains are highly conserved small protein modules involved with recognition of phosphothreonine epitopes [19]. They are present in a number of different protein classes including kinases, phosphatases and transcription factors. Some *in vitro* studies have suggested that the FHA domain of Ki-67 may be involved with recognition of mitotic phosphoproteins [20, 21], providing a possible explanation of how the FHA domain mediates clonogenic proliferation. Additional studies are needed to further elucidate the exact mechanism of how Ki-67 regulates CSC properties.

Our study also provides further explanation of Ki-67's predictive-prognostic paradox as described above. Ki-67's ability to regulate the CSC population may be key to understanding how cancers with high expression of Ki-67 can lead to a robust clinical response, yet still be associated with worse prognosis due to a short time to progression (Figure 6d). Indeed, several breast cancer studies of neoadjuvant (pre-surgical) endocrine therapy demonstrate that a decrease in Ki-67 expression is associated with effective response [3]. Similarly, a recent study demonstrated an improved prognostic value of Ki-67 when assessed after neoadjuvant chemotherapy in breast cancer patients [18]. Strikingly, patients who had a change from high to low Ki-67 expression after neoadjuvant chemotherapy had better disease-free survival than patients who had little change with chemotherapy, including patients whose primary tumors were initially characterized pre-therapy as having low-to-intermediate Ki-67 expression.

In summary, we have demonstrated via genome editing that Ki-67 is not necessary for cell viability or proliferation. However, our results strongly support that Ki-67 may act as a “licensing factor” to maintain the CSC niche. Given the number of recalcitrant cancers hallmarked by high expression of Ki-67, we feel that Ki-67 may prove to be a viable therapeutic target for anti-cancer drug development.

## MATERIALS AND METHODS

### Cell culture conditions

MCF-10A and derivative cells were propagated in standard growth medium consisting of DMEM:F12 (Invitrogen) supplemented with 5% horse serum (HS, Hyclone), 20ng/mL epidermal growth factor (EGF, Sigma), 10μg/mL insulin (Sigma), 0.5μg/mL hydrocortisone (Sigma), 0.1μg/mL cholera toxin (Sigma), and 1% penicillin/streptomycin (Invitrogen). For proliferation and growth arrest assays, phenol red-free DMEM:F12 medium and 1% charcoal dextran-treated FBS (CD) were used, and EGF was reduced to 0.2ng/mL for physiologic concentrations and 0ng/mL for growth arrest conditions. Standard growth media for DLD-1 and its derivative cells was McCoy's 5A medium (Invitrogen) supplemented with 5% fetal bovine serum (FBS, Hyclone) and 1% penicillin/streptomycin. All cells were maintained in a 37°C incubator with 5% CO_2_.

### Targeted knockout of MKI67

Targeting vectors were designed as previously described [22]. Briefly, homology arms were PCR-amplified from MCF-10A genomic DNA (gDNA) and ligated into the promoterless gene-targeting vector pSEPT. The homology arms and intervening neomycin selection cassette were then subcloned into an adeno-associated virus (AAV) vector backbone. After packaging in HEK-293T cells, targeting vectors were transduced into MCF-10A or DLD-1 cells, and antibiotic selection was performed using 120μg/mL and 500 μg/mL of G418 (Invitrogen), respectively. Neomycin-resistant colonies were expanded and screened for homologous integration of the targeting vectors via our previously described PCR-based method [23]. Colonies were then infected with an adenovirus encoding Cre recombinase to remove the selection cassette, followed by single-cell dilution and screening by PCR to confirm successful Cre recombination. Cloning and screening primers are listed in Supplemental Table 4.

### Proliferation assays

MCF-10A isogenic cells were seeded in triplicate at 1×10^3^ cells per well in growth arrest media overnight. The media was then changed to either standard growth or growth arrest media for the duration of the experiment. DLD-1 isogenic cells were seeded in the same manner, except standard growth media was used throughout. On specified days, cell viability was assessed by CellTiter-Glo according to the manufacturer's protocol.

### Clonogenic and chemotherapy assays

The isogenic MCF-10A and DLD-1 cells were plated at either 2.5×10^2^ or 1×10^3^ cells per well in 6-, 12-, 24-, and 48-well plates in standard growth media. The cells were allowed to form colonies for 10 days before being fixed and stained with 0.2% crystal violet (w:v) in 10% buffered formalin. Colony numbers were manually counted.

Single cell clonogenic assays for MCF-10A and DLD-1 cells were plated in five 96-well plates per clone at a density of 0.5 and 1.0 cells/well, respectively, in 175μL of standard growth media. Clonogenicity is scored by counting the number of wells with colonies per plate per clone, and time to colony formation along with relative colony size was also recorded.

For clonogenic assays in the presence of chemotherapeutic agents, cells were seeded at 5×10^4^ cells per well and treated the following day with 5-fluorouracil (5-FU, Sigma), cisplatin (Sigma), and doxorubicin in standard growth medium for 24 hours at doses ∼ IC50 for the parental cell lines. Following treatment, cells were plated in triplicate at 1×10^3^ in 6-well plates. On day 6 (MCF-10A) and day 10 (DLD-1), colonies were stained with crystal violet, solubilized with 1% SDS and measured for absorbance at 590nm.

### Immunohistochemical labeling and scoring of cancer tissues

Ki-67 proliferation index was assessed by immunohistochemistry (rabbit monoclonal antibody, clone 30-9, catalog number 790-4286, Ventana Medical Systems, Inc.,) on tissue microarrays (TMAs) containing 19 pairs of matched primary human invasive mammary carcinomas and their subsequent visceral metastases harvested at the time of surgery. TMAs contained 5-10 cores per tumor to minimize sampling error and have been previously described [24]. The TMAs were labeled by immunohistochemistry for Ki-67, and the average proliferation index across all cores was calculated for each case in increments of 5% nuclear labeling, from 0% to 100%. Labeling of benign breast lobules and lymphocytes served as positive internal controls.

If available, the Ki-67 proliferation index for the primary or metastatic tumor as reported in the surgical pathology report was also subsequently recorded and compared to the result on the TMA to ensure concordance. In four cases, the primary tumor Ki-67 was unevaluable on the TMA due to processing artifact; in these 4 cases, the reported Ki-67 proliferation index from the surgical pathology report was used for analysis. The remaining 14 cases had concordant Ki-67 proliferation indices in the primary tumor between the TMA result and the surgical pathology report. Ki-67 was rarely performed on the metastatic tumor in the surgical pathology report (n=4), but those 4 cases did have concordant values with the TMA.

### Western blot analysis

Whole-cell protein lysates were prepared from exponentially growing cells. Cells were resuspended in 150μL PBS (Invitrogen) supplemented with protease inhibitor (Roche Complete Mini) and 150μL 2X Laemmli sample buffer (Sigma) supplemented with protease inhibitor was then added, mixed and boiled for 10 minutes at 100°C. Protein lysates were resolved by SDS-PAGE on Novex 4% Tris-Glycine gels (Invitrogen), transferred to nitrocellulose membranes (Invitrogen), and probed with primary and horseradish peroxidase-conjugated secondary antibodies. Membranes were blocked and probed in 4% cold water fish skin gelatin (Sigma). The primary antibodies used in this study are anti-Ki-67 (MIB-1, Santa Cruz), anti-GAPDH (6C5, Abcam), anti-cyclin D1 (2922, Cell Signaling) and anti-cyclin E (M-20, Santa Cruz).

### Reverse phase protein array (RPPA)

Cells were harvested at ∼75-85% confluency using RPPA Lysis Buffer and 4X SDS Sample Buffer supplied by the RPPA Core Facility in MD Anderson Cancer Center. Cellular proteins were denatured by 1% SDS (with beta-mercaptoethanol) and diluted in five 2-fold serial dilutions in dilution buffer (lysis buffer containing 1% SDS). Serial diluted lysates were arrayed on nitrocellulose-coated slides (Grace Biolab) by Aushon 2470 Arrayer (Aushon BioSystems). 5808 array spots were arranged on each slide including the spots corresponding to positive and negative controls prepared from mixed cell lysates or dilution buffer, respectively. Each slide was probed with a validated primary antibody plus a biotin-conjugated secondary antibody. Only antibodies with a Pearson correlation coefficient between RPPA and western blotting of greater than 0.7 were used in reverse phase protein array study. The signal obtained was amplified using a Dako Cytomation–catalyzed system (Dako) and visualized by DAB colorimetric reaction. The slides were scanned, analyzed, and quantified using a customized-software Microvigene (VigeneTech Inc.) to generate spot intensity. Each dilution curve was fitted with a logistic model (“Supercurve Fitting” developed by the Department of Bioinformatics and Computational Biology in MD Anderson Cancer Center, “http://bioinformatics.mdanderson.org/OOMPA”). This fits a single curve using all the samples (i.e., dilution series) on a slide with the signal intensity as the response variable and the dilution steps as the independent variable. The fitted curve is plotted with the signal intensities – both observed and fitted - on the y-axis and the log2-concentration of proteins on the x-axis for diagnostic purposes. The protein concentrations of each set of slides were then normalized by median polish, which was corrected across samples by the linear expression values using the median expression levels of all antibody experiments to calculate a loading correction factor for each sample.

### Mouse xenografts

Parental DLD-1 cells and isogenic KOOKi-67 clones were propagated for three days prior to serial dilution and inoculation. For each group, ten 8- to 10-week-old female athymic nude mice (Taconic) were injected subcutaneously in the right flank with 200μL of 4:1 reduced growth factor Geltrex:HBSS (Invitrogen) containing serial dilutions of the parental and derivative DLD-1 cell lines. Tumor volumes were measured weekly and calculated by multiplying the length, width, and height of each tumor until reaching the upper limit of 1.5cm^3^. Tumors that reached a volume of 0.2cm^3^ were considered to have engrafted. The NIH Guide for the Care and Use of Laboratory Animals was followed in all experiments.

### Fluorescent *in situ* hybridization (FISH)

Exponentially growing cells were dissociated with trypsin and seeded at 3×10^4^ cells per well of an 8-chamber culture slide (BD Falcon) in standard growth conditions. After 48 hours, slides were pretreated using Paraffin Pretreatment Reagent Kits (Abbott) and dehydrated with graded ethanol concentrations. Vysis LSI 22 (BCR) SpectrumGreen and LSI EGFR SpectrumRed probes were applied to the slide, denatured at 95°C, and hybridized overnight at 37°C in a humidity chamber. Slides were then counterstained with 4′,6-diamidino-2-phenylindole (DAPI) before allele counting in approximately 200 cells per probe per clone under fluorescence microscopy.

### Flow cytometry

DLD-1 parental and KOOKi-67 clones were subjected to flow cytometry analysis for the expression of CD44 and CD133. Briefly, 2 million cells were harvested from 85% confluent flasks and resuspended in PBS with 0.1% BSA. The cells were washed and incubated at 4°C for 15 minutes with anti-CD44-allophycocyanin (APC) (1:20 dilution, clone G44-26, BD Biosciences) and anti-CD133-phycoerythrin (PE) (1:20 dilution, clone AC133, Miltenyi Biotec) antibodies, or mouse-specific IgG_2b_ ĸ-APC (1:100 dilution, BD Biosciences) and IgG_1_-PE (1:20 dilution, Miltnyi Biotec) antibodies. The cells were then washed and resuspended in PBS with 0.1% BSA and 2μg/mL propidium iodide (PI), and a FACSCalibur flow cytometer (BD Biosciences) was used for all analyses. The cells were first gated on the basis of side-scatter and forward-scatter, followed by the exclusion of nonviable (PI-positive) cells. The CD44^+^ and CD133+ gates were created on the basis of cellular staining with the isotype control antibodies (IgG_2b_ ĸ-APC and IgG_1_-PE, respectively).

### Quantitative Real Time RT-PCR Analysis

Total RNA was isolated and purified with the RNeasy Plus Mini Kit (Qiagen, Valencia, CA) following the manufacturer's protocol. cDNA was synthesized using a First Strand cDNA Synthesis Kit (Amersham Biosciences, UK) following the manufacturer's protocol. Quantitative real time RT-PCR was carried out using SsoAdvanced Universal SYBR Green Supermix (Bio-Rad, Hercules, CA) in conjunction with an iCycler (Bio-Rad, Hercules, CA) using the human PrimePCR Cytochemokines Tier 1 H96 and the human PrimePCR Growth Factors (SAB Target List) H96 plates (Bio-Rad, Hercules, CA). Amplification conditions were: 95°C for 2 min, followed by 40 cycles of 5 seconds at 95°C and 30 seconds at 60°C. cDNA templates were assayed in triplicate and results are presented at mean ± standard deviation (SD).

### Statistical analysis

All statistical analyses were performed using GraphPad InStat software (La Jolla, CA). A P value of less than 0.05 was considered significant.

## SUPPLEMENTAL MATERIALS FIGURES AND TABLES


