# Supplementary material for: Ki-67 is required for maintenance of cancer stem cells but not cell proliferation
Source: Oncotarget. 2016 Jan 28;7(5):6281–93. doi: 10.18632/oncotarget.7057 (PMC4868756; doi:10.18632/oncotarget.7057)
Supplement: Supplementary file 1 [file oncotarget-07-6281-s001.pdf]

Ki-67 is required for maintenance of cancer stem cells but not cell proliferation

Supplemental Materials

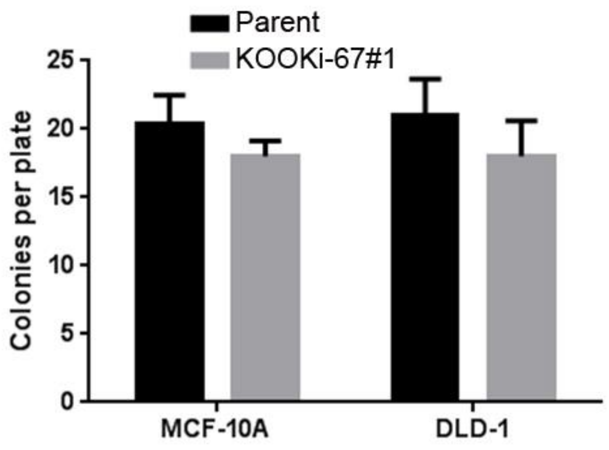

Supplemental Figure 1. Knock out of Ki-67 does not affect colony number. Parental MCF-10A, DLD-1 and derivative KOOKi-67 clones were seeded at 0.5 cell per well in 96-well plates and grown for 30 days under standard conditions, at which time the number of colonies per well were counted.

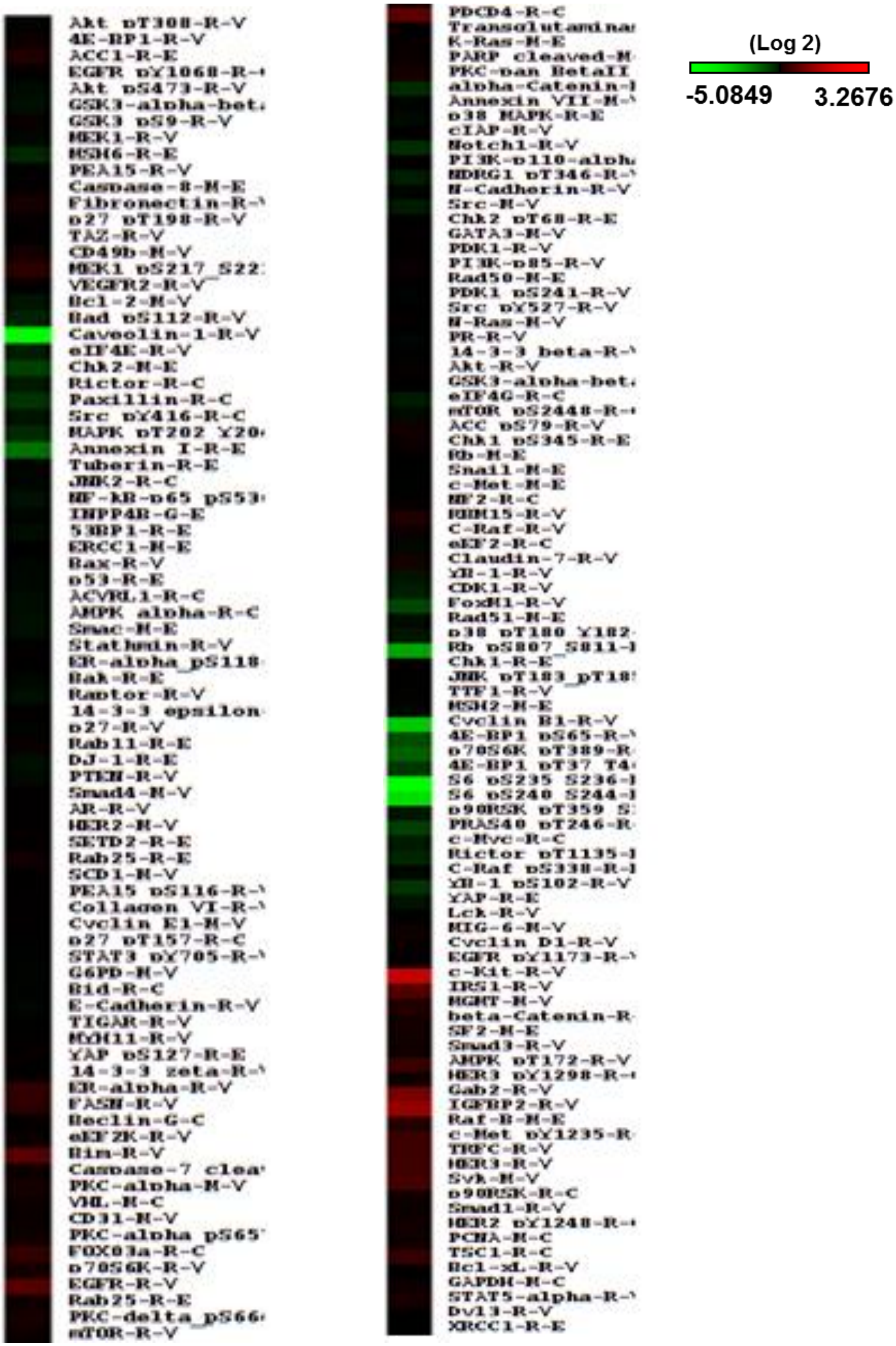

Supplemental Figure 2: **List of proteins expression analyzed by RPPA.** MCF-10A and DLD-1 parental and KOOKi-67 clones were subjected to RPPA analysis. DLD-1 cells were grown in standard full growth conditions while MCF-10A cells were grown in both full growth (+EGF) and growth arrest (-EGF) conditions.

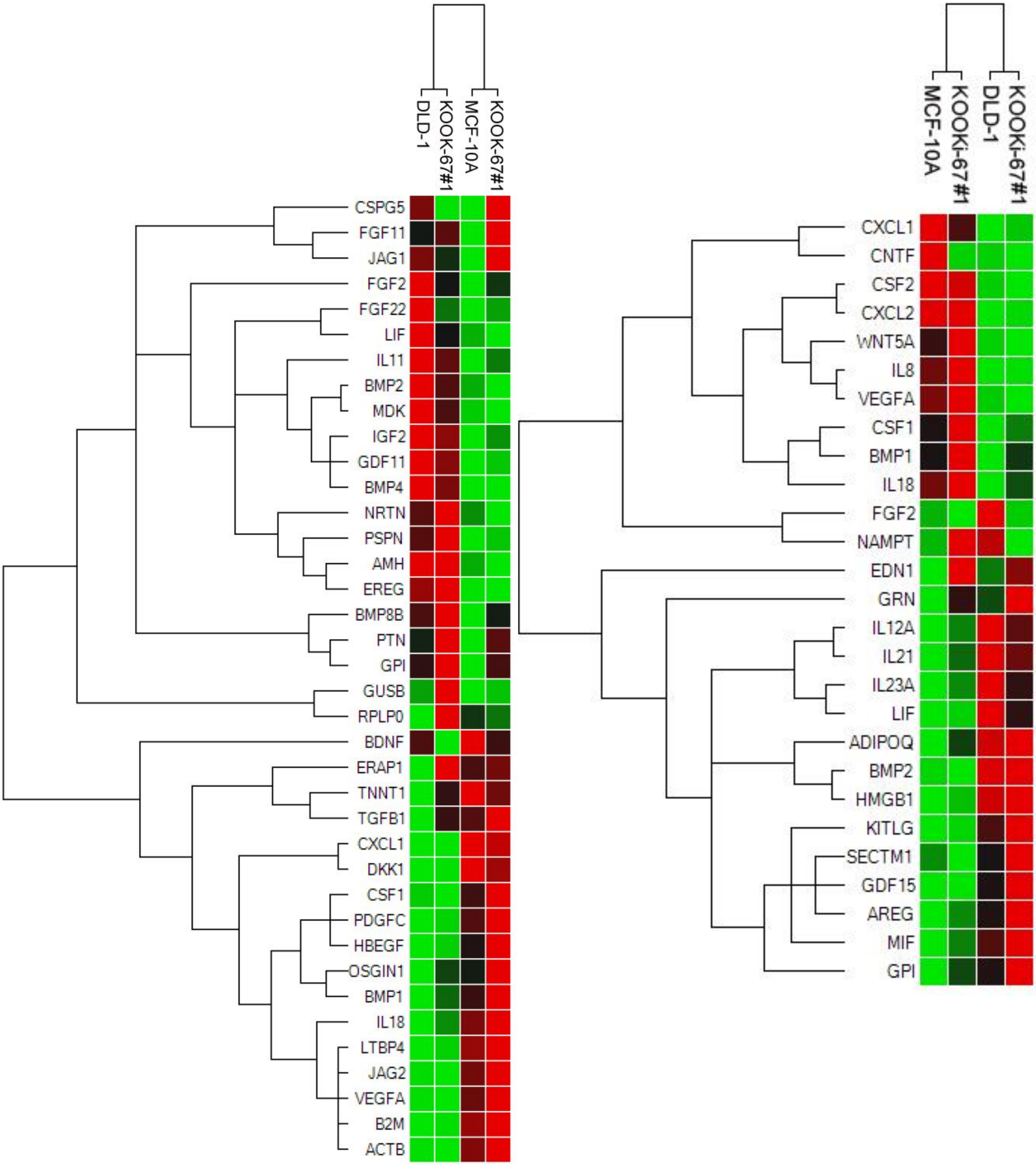

Supplemental Figure 3. Gene expression of growth factors and cytokines are similar between parental and KOOKi-67 cells. Quantitative PCR was performed using cDNA from parental and KOOKi-67 cells to assay for differences in growth factor and cytokine mRNA expression.

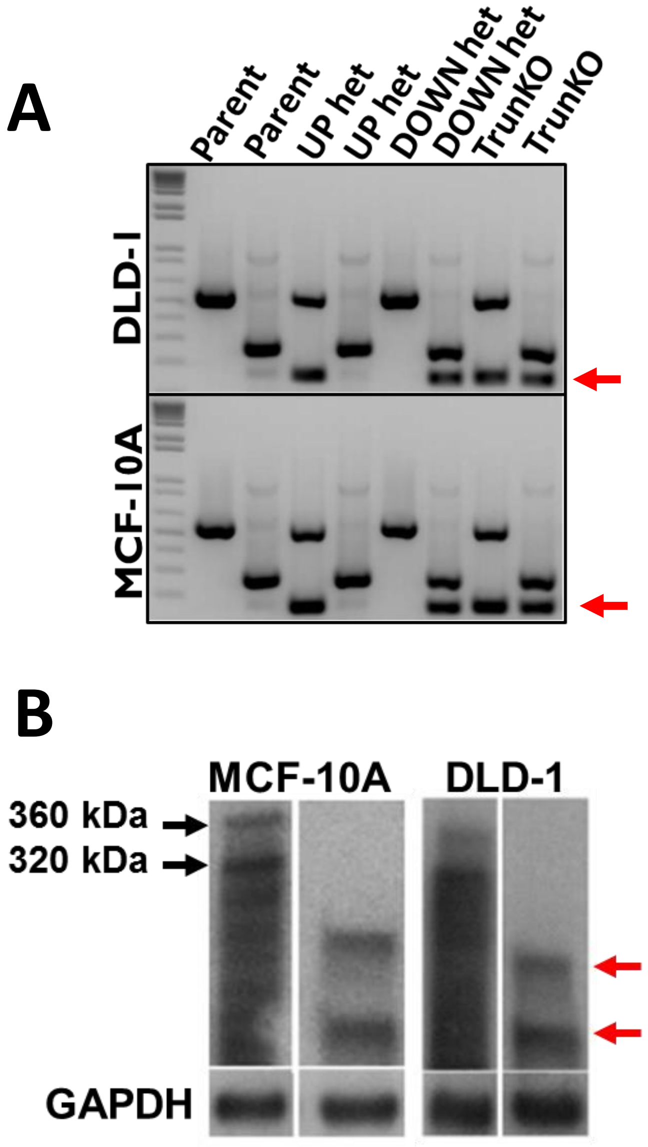

Supplemental Figure 4. PCR and western blot confirmation of TrunKO clones. A) PCR was performed using genomic DNA from parental and TrunKO cells to confirm correct targeting at genomic loci. Each genomic locus was assayed independently using primers specific for the targeted region. The presence of small molecular weight band (red arrow) confirmed targeting at the given locus. **B)** Western blot was performed using lysates from parental MCF-10A and DLD-1 cells (left lanes) and lysates from TrunKO clones (right lanes). Shown are the full length parental isoforms (360 kDa and 320 kDA), as well as the presence of truncated proteins (red arrows). GAPDH was used as a loading control.

Supplemental Table 1: Tumor engraftment is reduced by loss of Ki-67.

Tumor Engraftment					
		Number of cells per injection			
		10^6	10^5	10^4	10^3
Cell Line	Parent	10/10	10/10	9/10	8/10
	KOOKi-67 #1	10/10	9/10	8/10	6/10
	KOOKi-67 #2	10/10	9/10	8/10	6/10

Supplemental Table 2: Tumor engraftment is unaffected in Ki-67 TrunKO cells.

Tumor Engraftment					
		Number of cells per injection			
		10^6	10^5	10^4	10^3
Cell Line	Parent	20/20	20/20	19/20	18/19
	KOOKi-67 #1	10/10	9/10	8/10	7/10
	KOOKi-67 #2	10/10	9/10	4/5	4/5
	TrunKO	10/10	9/9	10/10	9/10

Supplemental Table 3. Cloning and screening primers used in this study.

KOOKi-67 Vectors	Cloning Primers			
	Region	Forward	Reverse	Screening
	5’HA	GATGCACCCAGGTATTTTCC	CCGCTCCTTTTGATAGTAACC	GTGCCCAGATGTTTGGTCC
	UP 3’HA	CACTTTCCCCTGAGCCTCAG	CACCTGATGCCTGAATGATGG	CTGAAGCTGTGGGGTTGG
	DOWN 3’HA	GCACAGAAGTGCTAGCATC	GCACCCAAATCCTTGAATTCC	CTCATGCAGTTTCAAAACAC
	Knockout Confirmation Screens			
	Region	Forward	Reverse	
	UP Allele	GGTTACTATCAAAAGGAGCGG	CACCTGATGCCTGAATGATGG	
	DOWN Allele	GGTTACTATCAAAAGGAGCGG	GATGCTAGCACTTCTGTGC	
	Deleted Region	GTGTCCCAATGTTTGGTCC	GGAAAGTGGGGACCGTCGAC	
TrunKO Vectors	Cloning Primers			
	Region	Forward	Reverse	Screening
	UP 5’HA	CCAGAACCAATAAACACCCC	TAGTTTTGCCAGCAGCCAC	CTCATGAAAGACACGGCACG
	UP 3’HA	AGCAGTAAGTGGTGAGAAAAAC	TAGCAGGTCCAGTTTCTCCAC	CGTCTGTGTGAGCTTGCCG
	DOWN 5’HA	CCAGTCAAAACCCCAACAAGC	TGCTGGTTTGGGTGTGTC	GAAGTATCCTGCAAATCTCC
	DOWN 3’HA	GACACCCCAGCAAGCACAAAG	GTGTGTGTGTGTGCTTTGCC	GCTGGGTTCCTCTTCTACTG
	Knockout Confirmation Screens			
	Region	Forward	Reverser	
	UP	GTGGCTGCTGGCAAAACTA	GTTTTTCTCACCACTTACTGCT	
	DOWN	GACACACCCAAACCAGCA	CTTTGTGCTTGCTGGGGTGTC	
